# Chemical and Biological Study of the Essential Oil Isolated from Fruits of *Citrus* x *limonia*

**DOI:** 10.3390/plants14050705

**Published:** 2025-02-25

**Authors:** Eduardo Valarezo, Jailene Paucar-Costa, Belén Morales-Guamán, Alfredo Caraguay-Martínez, Ximena Jaramillo-Fierro, Nixon Cumbicus, Miguel Angel Meneses

**Affiliations:** 1 Departamento de Química, Universidad Técnica Particular de Loja, Loja 1101608, Ecuador; afcaraguay@utpl.edu.ec (A.C.-M.); xvjaramillo@utpl.edu.ec (X.J.-F.); mameneses@utpl.edu.ec (M.A.M.); 2 Carrera de Bioquímica y Farmacia, Universidad Técnica Particular de Loja, Loja 1101608, Ecuador; gjpaucar1@utpl.edu.ec (J.P.-C.); abmorales6@utpl.edu.ec (B.M.-G.); 3 Departamento de Ciencias Biológicas y Agropecuarias, Universidad Técnica Particular de Loja, Loja 1101608, Ecuador; nlcumbicus@utpl.edu.ec

**Keywords:** chiral compounds, enantiomeric excess, Rutaceae family, (−)-(4S), (+)-(4R), limonene

## Abstract

*Citrus* x *limonia* is an aromatic species, known locally in Ecuador as *limón mandarina* or *limón chino*. In the present study, the chemical composition and biological activity of the essential oil isolated from this species were determined. The essential oil was extracted through hydrodistillation. The chemical composition and enantiomeric distribution of the essential oil were determined by gas chromatography. Antimicrobial activity was determined using the broth microdilution method against tree Gram-positive cocci, a Gram-positive bacillus, four Gram-negative bacilli, a fungus, and a yeast. The antioxidant activity was determined through ABTS (2,2′-azino-bis(3-ethylbenzothiazoline-6-sulfonic acid)) and DPPH (2,2-diphenyl-1-picrylhydrazyl) methods. The spectrophotometric method was used to determine anticholinesterase activity. In the essential oil, thirty-nine compounds were identified, which represented 99.35% of the total composition. Monoterpene hydrocarbons were the most representative group in terms of number of compounds (thirteen) and in terms of relative abundance (91.39%). The main constituents were found to be limonene (57.38 ± 1.09%), *γ*-terpinene (13.01 ± 0.37%), and *β*-pinene (12.04 ± 0.63%). Five pairs of enantiomers were identified in the essential oil from fruits of *Citrus* x *limonia*. The essential oil presented a minimum inhibitory concentration of 4000 μg/mL against *Aspergillus niger*. The antioxidant activity of essential oil was weak per the ABTS method, with a SC_50_ of 1.26 mg/mL. Additionally, the essential oil exhibited moderate anticholinesterase activity, with an IC_50_ of 203.9 ± 1.03 µg/mL. This study provides the first comprehensive analysis of the chemical composition and biological activities of the essential oil from fruits of *Citrus* x *limonia*.

## 1. Introduction

Essential oils (EOs) are complex blends of volatile compounds that can be found in different plant parts, including leaves, flowers, bark, roots, and fruits [1]. EOs have been valued for centuries in various cultures for their medicinal, culinary, and aromatic properties [2]. Recent research has highlighted the diversity of phytochemical compounds within essential oils, emphasizing their relevance in the pharmaceutical, cosmetic, and food industries [3]. Essential oils can be isolated from species of different botanical families such as Asteraceae, Lamiaceae, Myrtaceae, Rutaceae, and Verbenaceae [4].

The Rutaceae family, a prominent source of essential oils, comprises more than 158 genera and around 2013 species, primarily distributed across tropical and subtropical regions [5]. Within Rutaceae, the *Citrus* genus stands out not only for its edible fruits but also for the essential oils it produces, which hold high medicinal and commercial value. Named after the genus *Ruta*, the Rutaceae family is distinguished by plants with specialized glands for essential oil production [6].

*Citrus* fruits belong to the Aurantioideae subfamily within Rutaceae, a group further divided into two main tribes: Clauseneae, considered the more primitive, and Citreae. The Citreae tribe, containing 28 genera, is categorized into three subtribes: Triphasiinae, Balsamocitrinae, and Citrinae. Within the Citrinae subtribe are the “true citrus” genera, *Fortunella*, *Eremocitrus*, *Poncirus*, *Clymenia*, *Microcitrus*, and *Citrus*. These genera are recognized by their segmented fruit structure, filled with stalked, fusiform pulp vesicles, forming a unique structure known as hesperidia, which is not found in any other Rutaceae plants [7].

*Citrus* x *limonia* (L.) Osbeck [8,9] is a cultivated species which is found across various regions of Ecuador, is commonly found in the Galapagos, Coastal, and Andean areas, especially in the provinces of Loja, Esmeraldas, Galapagos, Imbabura, Los Ríos, and Pichincha [10]. The spices *Citrus* x *limonia* is characterized by thorny, semi-deciduous trees that are 3 to 6 m tall, with an open and irregular crown, a tortuous stem, and grayish bark. Its leaves are simple, alternate, and aromatic, with glands measuring 6 to 10 cm in length, and a non-winged petiole. The flowers are large, thin, and fragrant, gathered in axillary summits. Its fruits are of the berry type, ellipsoid in shape, generally with a small apical nipple, a slightly rough surface, and a yellow-green color, with few seeds [11]. Thriving at elevations of 0–3000 m [12], this species is valued for its high acidity and distinct orange-colored peel and pulp [13].

*Citrus* x *limonia* is a hybrid of mandarin (*Citrus reticulata*) and citron (*Citrus medica*), with a small genetic contribution from *Citrus micrantha*. In Ecuador, this species is known by the common names of *limón mandarina* or *limón chino*; however, worldwide, it is known by many other common names, such as *limón paraguayo* (Spanish), *limón misionero*, *lima mandarina*, *limón mandarino*, *limão capeta* (Portuguese), *laranja capeta*, *mandarin lime*, *lemandarin*, *rangpur lime* (English), and *sylhet lime* or *surkh nimboo* (Hindi). Some of these common names are also used to name the species *Citrus limonia* Osbeck, which is possibly a hybrid between *Citrus reticulata* and *Citrus aurantifolia*, *Citrus limon*, or *Citrus macrophylla* [7,14,15,16].

In general, species of the *Citrus* genus are known for their aromatic properties, with many species contributing essential oils to various industries such as pharmaceuticals, cosmetics, and food production [17,18]. The essential oils derived from *Citrus* species contain a variety of bioactive compounds, including monoterpenes such as limonene, *β*-pinene, and *γ*-terpinene. These essential oils are linked to antimicrobial, anti-inflammatory, antioxidant, anticarcinogenic, anthelmintic, insecticidal, larvicidal, and other properties [19,20,21]. Specifically, the presence of coumarins and flavonoids has been determined in the ethanolic extracts of *Citrus* x *limonia,* and the essential oil from this species has been shown to inhibit the growth of some microorganisms [11].

The essential oils isolated from *Citrus* have been studied abundantly; however, there are no studies on the essential oil extracted from the fruits of the *Citrus* x *limonia*. This fact motivated the realization of this study, with the aim of isolating and characterizing the physical properties and chemical profile of the essential oil from fruit of *Citrus* x *limonia*. Furthermore, this research will examine the biological activity of the essential oil, focusing on its antimicrobial, antifungal, antioxidant, and anticholinesterase activities. This research is based on the hypothesis that the essential oil of this species, being of the *Citrus* genus, will present characteristic compounds such as limonene and activities such as antibacterial and antioxidant commonly attributed to species of this genus.

## 2. Results

### 2.1. Essential Oil Isolated

A total of 10.84 kg of fruits from *Citrus* x *limonia* with a moisture level of 76.68 ± 2.27% *w*/*w* (fresh) were hydrodistilled in three different batches for the purpose of isolating the EO. From this plant material, 87 mL of EO was obtained, corresponding to a yield of 0.79 ± 0.11% (*v*/*w*) or 7.9 ± 1.1 mL/kg.

### 2.2. Physical Properties of Essential Oil

The essential oil from fruits of *Citrus* x *limonia* was presented as an unctuous liquid of subjective mustard green color, which was less dense than water. Table 1 shows the mean values of physical properties of EO; additionally, the standard deviation (SD) is shown.

### 2.3. Chemical Composition of Essential Oil

Table 2 shows the compound number (CN), retention time (RT) at which each the compound elutes, retention indices calculated (RICs), retention index obtained from the literature (RIR), relative abundance (%) with its standard deviation (SD) group to which each compound belongs (GC), chemical formula (CF), and monoisotopic mass (MM). Thirty-nine compounds were identified in EO from fruits of *Citrus* x *limonia*, representing 99.35% of the total composition. The compounds were classified into 5 groups, according to the number of carbons (monoterpenes: 10 carbons; sesquiterpenes: 15 carbons) and the presence of oxygen (oxygenated and non-oxygenated), and other compounds (non-terpenic compounds). The monoterpene hydrocarbon (MH) group was the most representative in the number of compounds (thirteen) as well as in terms of relative abundance (91.39%). In fact, the three main compounds belong to this group. No compounds belonging to the diterpene (oxygenated and non-oxygenated) group were identified. The main constituents (>5%) in terms of abundance were found to be MH (CF: C_10_H_16_; MM: 136.13 Da) limonene (compound number, CN: 10) with an abundance of 57.38 ± 1.09%, *γ*-terpinene (CN: 44) with 13.01 ± 0.37%, and *β*-pinene (CN: 5) with 12.04 ± 0.63%.

### 2.4. Enantiomeric Analysis

Using chiral columns, it was possible to separate four pairs of enantiomers in the EO from fruits of *Citrus* x *limonia*. Table 3 shows the retention time (RT), retention index (RI), enantiomeric distribution (ED), and enantiomeric excess (e.e.) for each pair of compounds. For limonene, one of the four enantiomers is found to be almost pure, with an e.e. percentage of 96.14%, although it was not possible to determine which one.

Table 4 shows the results obtained using the MEGA-DEX DAC Beta enantioselective stationary phase column. This column was able to separate a pair of enantiomers.

### 2.5. Antibacterial Activity

Table 5 shows the minimum inhibitory concentration (MIC) values of the EO from fruits of *Citrus* x *limonia*, both the positive control and the negative control. At the maximum concentration tested (4000 µg/mL), it was not possible to determine the MIC value against any bacteria. All microorganisms showed normal growth in negative control.

### 2.6. Antifungal Activity

Table 6 shows the values for the minimum inhibitory concentration (MIC) of the EO from fruits of *Citrus* x *limonia* against two fungi and yeasts, the positive control and the negative control. The essential oil showed weak activity against *Aspergillus niger* with a MIC of 4000 µg/mL

### 2.7. Antioxidant Activity

Table 7 shows the scavenging capacity (SC_50_) values expressed in µg/mL of the EO for ABTS and DPPH and the antioxidant capacity in µM/g for the trolox equivalent antioxidant capacity (TEAC) values.

### 2.8. Anticholinesterase Activity

**Figure 1:**
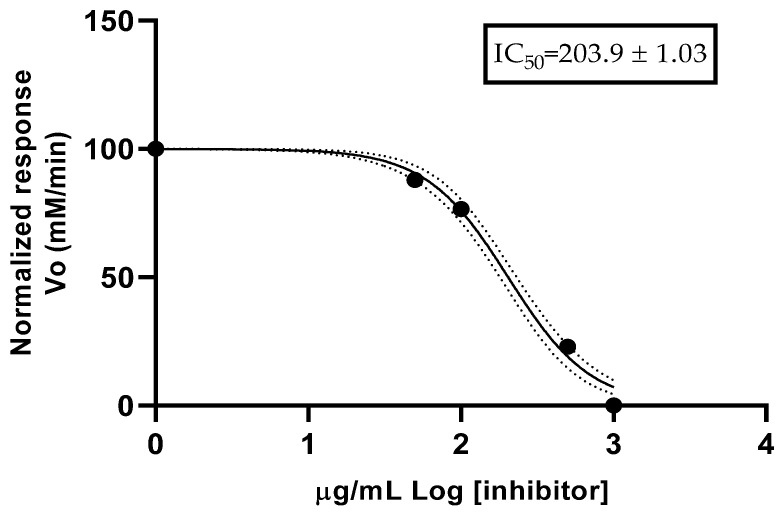
Anticholinesterase activity of essential oil from fruits of *Citrus* x *limonia*.

Figure 1 shows the rate of the reaction curve for anticholinesterase (anti-AChE) activity. The EO from fruits of *Citrus* x *limonia* exhibited a half-maximal inhibitory concentration (IC_50_) value of 203.9 ± 1.03 µg/mL. The positive control (donepezil) reported an IC_50_ value of 4.71 ± 0.51 µg/mL.

## 3. Discussion

The extraction of essential oil from fruits of *C.* x *limonia* shows a yield of 0.79 ± 0.11% (*v*/*w*). It is known that the extraction yield depends on the origin of the vegetal species, the location, farming practices, and harvest season. In the literature, different values of extraction yield have been published for the essential oil of *C. limonia* Osbeck peels or barks: Jing et al. [22] reported 1.14 ± 0.01% (*v*/*w*) by steam distillation, Barros Gomes et al. [23] reported 2.54% by hydrodistillation, and Millezi et al. [24] reported 1.85% (*w*/*w*). In addition, Jing et al. [22], in 2015, also reported the extraction yield of essential oils from fruit peels of several citrus species, with values ranging from 1.84% to 0.38% (*v*/*w*).

Most essential oils have densities lower than water. In this study, the density was 0.8717 ± 0.0001 g/cm^3^. The density is related to the lipophilic nature of the chemical compounds present in the essential oil. Barros Gomes et al. [23], in 2020, reported a density of 0.842 g/mL for EO from bark of *C. limonia* Osbeck. The color is a qualitative measure and a fast quality indicator of the purity of compounds; its variations could be correlated to thermal stress during the extraction. The refractive index, optical activity, and chemical composition of an essential oil are related since they depend on the molecular structure and the proportion of its components. The refractive index measures the deflection of light when passing through the oil; it is influenced by the electron density and the nature of the compounds present, which is why it is used as a quality and purity indicator in essential oils. Optical activity, on the other hand, is due to the presence of chiral molecules, such as monoterpenes and sesquiterpenes, which can rotate the plane of polarized light in opposite directions. However, enantiomers of the same compound can have the same refractive index, but opposite optical activity. The chemical composition of the oil defines both the refractive index and the optical activity, as different proportions of hydrocarbons, alcohols, esters, and ketones affect both parameters. For example, (R)-(+)-limonene and (S)-(−)-limonene have similar refractive indices (~1.47), but opposite optical activities, demonstrating that the combination of these analyses is essential in the characterization and authentication of EO [25]. To the best of our knowledge, there are no reports of the physical properties of the essential oil of *C.* x *limonia*. However, with comparison purposes, some values could be included: Mushtaq et al. [26] reported a refractive index between 1.464 and 1.472, and an optical rotation between 76.666 and 93.257 for various citrus fruits. Conversely, Rajat et al. [27], in 2024, reported a refractive index of 1.44 ± 0.0008 for the EO of *C. macroptera*. These physical properties and the chemical composition help identify and characterize the quality and purity of EOs. In addition, the evaluation of these measures along the time could be used as an indicator of stability or for detecting alterations due to oxidation or light effects, which is highly relevant in industrial applications.

The identification and quantification of chemical compounds in the EO from fruits of *C.* x *limonia* showed that the most representative group was monoterpene hydrocarbons, containing the main compounds: limonene 57.38 ± 1.09%, *γ*-terpinene 13.01 ± 0.37%, and *β*-pinene 12.04 ± 0.63%. The chemical composition of citrus peel essential oils is complex and rich in limonene (30% to 70% in different varieties) along with other significant components: *α* and *β*-pinene, *γ*-terpinene, terpinolene, sabinene [19]. Some studies show differences in the chemical composition, as such reported by Minh Tu et al. [28] for the EO from peel of *C. limonia* Osbeck, with content of limonene lower than other citrus species, whereas *γ*-terpinene, *β*-pinene, and *α*-pinene occurred in higher proportions. Jing et al. [22] reported R-(+)-limonene (64.09%), para-mentha-3,8-diene (21.26%), and *α*-pinene (4.30%) as the main components in the essential oil of *C. limonia* Osbeck fruit peels, whereas *γ*-terpinene and *β*-pinene were not found; furthermore, they reported that the predominant components for 20 citrus species/varieties were R-(+)-limonene (55.05–91.06%) and para-Mentha-3,8-diene (0.04–28.04%). Similarly to our findings, Amorin et al. [20], in 2016, reported the chemical composition of EO from peel of *C. limonia*, where the main compounds are limonene 65.7% and *γ*-terpinene 12.3%. Abdel-Daim et al. [29], in 2020, reported the main compounds to be limonene D (20.38%), 5-methyl-pentadecane (5.33%), (n)-menthol, (5.18%), 3,7-dimethyl-(E)-2,6-octadienal (5.16%), 3,7-dimethyl-2,6-octadienal (4.92%), and nonadecane (4.40%). Barros Gomes et al. [23], in 2020, analyzed the chemical compounds present in EO from the bark of *C. limonia* Osbeck and found that the major compounds were limonene (44.75%), *β*-pinene (23.01%), and *m*-cymene (13.55%). The differences in chemical composition are related to the citrus species, the postharvest treatment, the part of the plant used, the extraction method, among others. Differences in chemical composition are observed even between the same species, as reported by Acevedo et al. [30], who determined the chemical profile of oils from peel of Rangpur lime (*Citrus limonia*) from three lime populations. Moreover, Lota et al. [31] identified four chemotypes in the peel essential oil of limes: limonene; limonene/*γ*-terpinene; limonene/*β*-pinene/*γ*-terpinene; and limonene/*γ*-terpinene/*β*-pinene/oxygenated products.

This is the first report on the enantiomeric analysis of the essential oil from peels of *C.* x *limonia*. Four enantiomers were identified with the MEGA-DEX DET Beta column from these compounds. For *α*-pinene, sabinene, and limonene, it was not possible to determine which of the two enantiomers corresponds to the excessive compound. One of the enantiomers for limonene (98.07%) and *α*-pinene (96.08%) was present almost pure. For *β*-pinene, (+)-(1R,5R)-*β*-pinene was present in excess, at 70.84%, while sabinene was a racemic mixture. When the MEGA-DEX DAC Beta column was used, *α*-terpineol was identified as the only enantiomeric pair. The excessive compound was present at 82.05% enantiomeric distribution; however, it was not possible to determine to which one it corresponds.

The essential oil did not show antimicrobial activity at the concentrations evaluated. For all the microorganisms, the MIC was higher than 4000 µg/mL. There are no reports about the antimicrobial activity of EO from fruit or peels of *C.* x *limonia*. As a means of comparison, some scientific reports are mentioned. Chinataluri et al. [32] reported the antimicrobial activity of essential oils from various citrus species by disc diffusion method, with *C. maxima* and *C. aurantifolia* showing potential antimicrobial properties against *Acinetobacter baumannii*, while *Enterococcus faecalis* and *Pseudomonas aeruginosa* were resistant organisms. For *C. limonia*, Oliveira et al. [33], in 2022, reported activity against *S. aureus* with an MIC of 62.4 µg/mL and *Listeria monocytogenes* with an MIC of 400 µg/mL.

The antagonist effect of the chemical compounds contained in the EO could reduce the effectivity of individual compounds, such as limonene, the main compound in the EO of citrus species, which has been reported with antimicrobial activity against *Yersinia enterocolitica* and *S. aureus* [34]. Lin et al. [35] also reported a review of the antimicrobial effect of D-limonene, showing antibacterial activity against *Escherichia coli* (ATTC 8739, MIC: 1 µg/mL), *Staphylococcus aureus* (ATCC 29523, MIC: 3000 µg/mL), and *Enterococcus faecalis* (MIC: 12.5 mg/mL) as well as antifungal activity against *Candida albicans* (ATCC 90028, MIC: 300 µg/mL).

The antioxidant activity is a common analysis in bioprospective studies to determine potential applications of bioactive compounds or ingredients. The applications are related to preventing illnesses linked with oxidative damage caused by reactive oxygen species, such as Alzheimer, Parkinson, cardiovascular diseases, among others. The EO from fruits of *C.* x *limonia* showed weak antioxidant activity, expressed as SC_50_. The SC_50_ value was 1.26 mg/mL for ABTS, and 8.14 mg/mL for DPPH, while the TEAC was 21.75 ± 3.75 µM TE/g. Budiarto et al. [36], in 2024, reported a significantly reduced impact on antioxidant capabilities of lemon EO’s compared with the control group. Limonene has been reported to show antioxidant activity at concentrations of 2–2000 µM in the ABTS assay [37]. On the other hand, Himed et al. [38], in 2019, reported an EO value of SC_50_ 0.66 mg/mL in the DPPH assay for lemon peels. The antioxidant properties of the essential oil vary with the chemical composition and correspond to the synergistic relationship between major and minor components.

Regarding the anticholinesterase (anti-AChE) activity, the OE from fruits of *C.* x *limonia* showed an IC_50_ of 203.9 ± 1.03 µg/mL in anti-AChE activity assay, a value superior to the positive control (donepezil). There are no reports for anti-AChE activity of the EO from fruits of *C.* x *limonia*; however, other results for citrus species could be mentioned. Aaza et al. [39] mentioned a low activity for limonene, with an IC_50_ of 0.5863 mg/mL, with it being the main compound in *E. globulus* essential oil, while, for *C. limon*, there was an IC_50_ of 0.8499 mg/mL. For the EO of *C. limonia,* Oliveira et al. [33] reported an AChE activity with an IC_50_ value of 128.7 µg/mL, while, for EOs of other *Citrus* species, the IC_50_ reported from 95.9 µg/mL for *C. deliciosa* to 142.3 µg/mL for *C. latifolia*. The study of anticholinesterase activity aims to identify potential applications of essential oils for the treatment of Alzheimer’s disease. For this purpose, Santos et al. [40], in 2018, reported a review of different studies and presented a classification with three categories depending on the IC_50_ value for Ellman’s method: high potency of IC_50_ < 20 µg/mL, moderate potency of 20 < IC_50_ < 200 µg/mL, and low potency of 200 < IC_50_ < 1000 µg/mL. Based on this scale, the essential oil from fruit of *C.* x *limonia* presented low AChE potency.

Despite the weak antifungal, antibacterial, and antioxidant activity, as well as the moderate anticholinesterase activity, of the essential oil isolated from fruits of *Citrus* x *limonia* determined in this study, the biological properties attributed to the main compounds of this essential oil are diverse in the literature. Limonene and *β*-pinene have demonstrated anti-inflammatory activity as they have shown the ability to modulate the production of proinflammatory cytokines [41,42]. These monoterpenes show analgesic effects since they can interact with pain receptors, decreasing nociceptive perception [43]. *γ*-Terpinene showed an antihyperalgesic effect in a model of neuropathic pain induced by tumor cells [44]. Limonene also acts as a neuroprotector since it has been shown to reduce oxidative damage and modulate neurotransmitters in models of neurodegenerative diseases [45]. A specific enantiomer of limonene, (R)-(+)-limonene, has shown anxiolytic effect [46]. Another relevant activity attributed to these monoterpenes is the repellent and insecticidal effect, which makes them useful in biopesticide applications [47]. In addition, these compounds have cytotoxic and antitumor activity, inhibiting cell proliferation and inducing apoptosis in cancer cells [48].

## 4. Materials and Methods

### 4.1. Materials

Aliphatic hydrocarbons, used in calibration curve in GC-FID, were acquired from ChemService (West Chester, PA, USA). Helium was purchased from INDURA (Quito, Ecuador). For the antimicrobial analysis, the Sabouraud dextrose broth, fluid thioglycollate medium, Mueller–Hinton broth, and Mueller–Hinton II broth were acquired from DIPCO (Quito, Ecuador), while phosphate-buffered saline (PBS), and tris hydrochloride (Tris-HCl), were purchased from Sigma-Aldrich (San Luis, MO, USA). For the bioactive analysis, 2,2-diphenyl-1-picrylhydryl (DPPH), 2,2′-azinobis-3-ethylbenzothiazoline-6-sulfonic acid (ABTS), 5,5′-dithiobis (2-nitrobenzoic acid) (DTNB), acetylcholinesterase (AChE), acetylthiocholine (AcSCh), butylated hydroxytoluene (BHT), dichloromethane (DCM), donepezil, dimethyl sulfoxide (DMSO), methanol (MeOH), magnesium chloride hexahydrate, sodium sulfate anhydrous, and trolox were purchased from Sigma-Aldrich (St. Louis, MO, USA). Reagents of analytical grade were used directly without any additional purification.

### 4.2. Plant Material

The fruits of *Citrus* x *limonia* were collected at La Florida, canton Palanda, province of Zamora Chinchipe. The site of collection is located at an altitude of 1390 m.a.s.l., at 4°37′42.9″ south longitude and 79°07′44.4″ west latitude. Once the fruits were collected, they were transported in airtight plastic containers to the university facilities. The identification of the plant material was made by botanist Nixon Cumbicus. A voucher (code HUTPL15058) was deposited at the Herbarium of Universidad Técnica Particular de Loja.

### 4.3. Postharvest Treatments

The fruit samples arrived four hours after being collected and were immediately subjected to postharvest treatment. This treatment involved the removal of foreign or deteriorated fruit samples.

### 4.4. Moisture Determination

The humidity of *Citrus* fruit samples was determined using Equation (1), and an analytical balance (Mettler AC 100, Mettler Toledo, Columbus, OH, USA), according to the method AOAC 930.04-1930 entitled Loss on dried (Moisture) in plants.

(1) $$Moisture\left(\%\right)=\frac{wi-wo}{wi}\times 100$$

where w is weight sample of “i” initial and “o” after drying.

### 4.5. Essential Oil Isolation

The patented device (TITLE No. PI-2022-012) called “Device for the release of essential oil from a plant matrix by crushing by immersion centrifugal force” was used. Accordingly, the plant material was treated for 50 s. It was immediately hydrodistilled using a Clevenger-type apparatus (80 L distiller, local construction). The process began by adding 16 L of water to the distiller, followed by the *Citrus* fruit samples. Extraction was conducted for 3 h, from the collection of the first drop of distillate. The resulting vapor, containing the EO and water, was condensed, and the OE was separated by decantation. To dry the oil, anhydrous sodium sulfate was used. The dried EO was then stored at 4 °C in sealed amber vials.

### 4.6. Determination of the Physical Properties of the Essential Oil

Three objective physical properties of the EO were determined: density, refractive index, and optical rotation, as well as a subjective physical property, color. The density of the EO was measured following the AFNOR NF T 75-111 standard (equivalent to ISO 279:1998 [49]) using an analytical balance (Mettler AC 100, Mettler Toledo, Columbus, OH, USA). The refractive index was determined following the AFNOR NF T 75-112 standard (similar to ISO 280:1998 [50]) using a refractometer (model ABBE, BOECO, Hamburg, Germany). The optical rotation was measured in accordance with ISO 592:1998 [51], using an automatic polarimeter (Mrc-P810, MRC, Holon, Israel). The color of the EO, considered a subjective property, was obtained using the PINETOOL website https://pinetools.com/ (accessed on 13 November 2024), where a photograph of the EO with a white background had been uploaded. All measurements were conducted at a constant temperature of 20 °C.

### 4.7. Identification of Essential Oil Compounds

#### Quantitative and Qualitative Analysis

The chemical compounds present in the EO from fruits of *C.* x *limonia* were identified using gas chromatogry (GC) (Thermo Scientific, Trace 1310, Waltham, MA, USA) provided with Thermo Scientific Chromeleon 7.2 Chromatography Data System (CDS) software and NIST 17 and AMDIS 2.7 mass spectral libraries database. For the quantitative analysis, the chromatograph was coupled to a flame ionization detector (GC-FID), whereas, for the qualitative analysis, it was coupled to a quadrupole mass spectrometer (GC-MS) (ISQ 7000, Thermo Scientific, Waltham, MA, USA). In both cases, an automatic injector (AI 1310, Thermo Scientific, Waltham, MA, USA) and a nonpolar GC column (TR-5MS, Thermo Scientific, Waltham, MA, USA) with stationary phase 5%-phenyl-methylpolyxilosane, 0.25 µm of stationary layer thickness, 0.25 mm of diameter, and 30 m of length were used. A total of 1 µL was injected at a 1/100 *v*/*v* (EO/DCM) dilution with a split ratio of 1:50. Helium was used as the carrier gas at a constant flow rate of 1.0 mL/min, and with an average velocity of 25 cm/s for the quantitative analysis, while, for the qualitive analysis, the flow rate was 0.9 mL/min, with an average velocity of 34 cm/s. The injector and detector temperatures in both analyses were set to 230 °C. The temperature ramp in the oven was similar in both analyses: 50 °C for 3 min, from 50 °C to 230 °C at 3 °C/min (60 min), and 230 °C for 3 min (total 66 min). The relative amounts of the compounds were calculated based on the GC-FID peak areas, without applying a correction factor. For mass spectrometry (MS) analysis, the following parameters were used: mass range of 40 to 350 *m*/*z*, electron multiplier 1600 eV, ionization energy 70 eV, and scan rate of 2 scan/s. The retention index (RI) of each compound was calculated using Equation (2) [52]:

(2) $$RI=100C+100\frac{\left(RTx-RTn\right)}{\left(RTN-RTn\right)}$$

where C is the carbon number of aliphatic hydrocarbon that elutes before the compound of interest. RTx is the retention time of the compound of interest. RTn and RTN are the retention times of the aliphatic hydrocarbons that elute immediately before and after the compound of interest, respectively.

Compounds were identified by comparing their RI and mass spectra with published data [53].

### 4.8. Enantioselective Analysis

The enantiomeric analysis of the EO from fruits of *C.* x *limonia* was performed using the same chromatograph, detector, and injector as described for the qualitative analysis. To determine the enantiomeric distribution, two enantioselective GC columns of 30 m length, 0.25 mm in internal diameter, and with 0.25 μm thickness stationary phase were used: one of which was called MEGA-DEX DET-Beta (Mega, Legnano, Italy) with diethyl tertbutylsilyl-beta-cyclodextrin stationary phase, and the other was called MEGA-DEX DAC-Beta (Mega, Legnano, Italy) with diacetyl tertbutylsilyl-beta-cyclodextrin stationary phase. The split ration, gas carrier, flow velocity, injector and detector temperatures, temperature ramp, and MS parameters were the same as those described for the qualitative analysis. The elution order of the compound enantiomers was determined based on column’s technical specifications. The enantiomeric excess (e.e.) was calculated as the difference between the percentage of the major enantiomer and that of the minor enantiomer.

### 4.9. Antimicrobial Activity

#### 4.9.1. Determination of Antibacterial Activity

The antibacterial activity of the EO from fruits of *C.* x *limonia* was tested against *Enterococcus faecalis* (ATCC 19433), *Enterococcus faecium* (ATCC 27270), and *Staphylococcus aureus* (ATCC 25923) as Gram-positive cocci, against *Lysteria monocytogenes* (ATTC 19115) as Gram-positive bacillus, and against *Escherichia coli* O157:H7 (ATCC 43888), *Campylobacter jejuni* (ATCC 33560), *Pseudomonas aeruginosa* (ATCC 10145), and *Salmonella* enterica subs enterica serovar *Thypimurium* WDCM 00031, derived from (ATCC 14028) as Gram-negative bacilli. The method was previously described by Valarezo et al., 2021 [54]. In summary, a 96-microwell plate was used for the antibacterial assay, with assaying concentrations of the EO ranging from 4000 to 15.62 µg/mL through two-fold serial dilution. For *Campylobacter jejuni*, decreasing concentrations of EO from 4000 to 31.25 µg/mL were used [55]. Different substances were used as a positive control: ampicillin for Gram-positive cocci and ciprofloxacin for Gram-positive bacillus and Gram-negative bacilli. As a negative control, DMSO at 5% was used, while the maximum concentration of EO was 4000 µg/mL. The MIC (minimum inhibitory concentration), defined as the lowest concentration of an antimicrobial which inhibited the growth of a microorganism after its incubation, was used to report the antibacterial activity values.

#### 4.9.2. Determination of Antifungal Activity

The antifungal activity of the EO from fruits of *C.* x *limonia* was carried out according to the method described by Valarezo et al., 2021 [54]. The antifungal activity was tested against the fungus *Aspergillus niger* (ATCC 6275) and the yeast *Candida albicans* (ATTC 10231). In summary, a 96-microwell plate was used for testing the antifungal activity at concentration of the EO ranging from 4000 to 15.62 µg/mL, through two-fold serial dilution; the antifungal activity was reported as MIC. The final concentration of spores was 5 × 10^4^ spores/mL. The EO was dissolved in SDB with fungal inoculum to acquire the required concentrations. The maximum evaluated concentration was 4000 µg/mL; amphotericin B was used as a positive control, and DMSO as a negative control.

### 4.10. Evaluation of Antioxidant Capacity

#### 4.10.1. ABTS Radical Cation Scavenging Activity

The free radical scavenging activity of the EO from fruits of *C.* x *limonia* was determined using the ABTS method, also known as the trolox equivalent antioxidant capacity (TEAC) method [56]. This method involves the generation of the ABTS radical cation (ABTS^•+^) through oxidation. The assay was conducted following the procedure described by Valarezo et al., 2021 [57], utilizing a colorimetric approach, and a UV-Vis spectrophotometer (Genesys 10S UV-Vis Spectrophotometer, Thermo Fisher Scientific, Waltham, MA, USA). The antioxidant activity of the EO was assessed by measuring the reduction in ABTS^•+^ at 734 nm. Antiradical capacity was expressed as the half-scavenging concentration (SC_50_) calculated from the dose–response curve. The maximum tested concentration was 2000 µg/mL. Trolox was used as a positive control, while MeOH served as a negative control. Using the graph of % EO Inhibition vs. the Logarithm (Log10) of the concentration, the IC_50_ (concentration at which the % Inhibition is equal to 50%) of the essential oil was calculated. The TEAC value is obtained from the relationship between the IC_50_ of trolox in µM/mL and the IC50 of the sample in g/mL.

#### 4.10.2. DPPH Radical Scavenging Activity

The free radical scavenging activity of the EO from *C.* x *limonia* fruits was also evaluated using the DPPH assay. In this method, the DPPH reagent generates the DPPH radical (DPPH^•^). The assay was conducted following the procedure described by Valarezo et al., 2021 [57]. The maximum evaluated concentration was 10,000 µg/mL. The equipment and the negative and positive controls were the same as those used in the ABTS assay, with the difference being that the antiradical capacity of EO was assessed by measuring the reduction in DPPH^•^ at 515 nm.

### 4.11. Determination of Anticholinesterase Activity

Anticholinesterase activity was evaluated based on the method described by Ellman et al. [58], following the protocol detailed by Valarezo et al., 2022 [55]. The assay was performed using a microplate spectrophotometer (EPOCH 2, BioTek, Winooski, VT, USA). Briefly, reaction mixtures containing s50 mM Tris buffer (pH 8.0), acetylthiocholine, Ellman’s reagent (DTNB), and the EO at decreasing concentrations were pre-incubated at 25 °C for three minutes. The reaction was initiated by adding acetylcholinesterase, and its progression was monitored at 412 nm. The IC_50_ (half inhibitory concentration) was determined using a non-linear regression model (normalized response vs. log Inhibitor-variable slope). Donepezil served as the positive control, while MeOH was used as the negative control.

### 4.12. Statistical Analysis

The data were collected on a Microsoft Excel spreadsheet and analyzed using Minitab 17 (Version 17.1.0., Minitab LLC, State College, PA, USA) to calculate measures of central tendency and standard deviation. The evaluation of antioxidant capacity and anticholinesterase activity was analyzed using GraphPad Prism, version 6.0 (GraphPad Software Inc., San Diego, CA, USA). All procedures, including essential oil isolation, determination of physical properties, antioxidant capacity evaluation, and anticholinesterase activity assessment were performed in triplicate. The identification of EO compounds, enantioselective analysis, and antimicrobial activity assays were conducted in nine replicates.

## 5. Conclusions

The chemical composition, enantiomeric distribution, and antibacterial, antifungal, antioxidant, and anticholinesterase activities of essential oil from fruits of *Citrus* x *limonia* were determined for the first time. Thirty-nine chemical compounds were identified with limonene ((−) and (+) enantiomers) being the main compounds. *Citrus* x *limonia* essential oil exhibited weak antioxidant activity and low anticholinesterase activity. This research enhances the understanding of aromatic plants of Ecuador. It establishes a foundation for future investigations into the biological properties, aromatic profiles, and potential applications of the enantiomers identified in the essential oil of *Citrus* x *limonia*. Additionally, future studies are encouraged to explore the anti-inflammatory activity of this essential oil.

## Figures and Tables

**Table 1:** Physical properties of the essential oil from fruits of *Citrus* x *limonia*.

	*Citrus* x *limonia* Fruit EO	
	Mean	SD
Density, *ρ* (g/cm^3^)	0.8717	0.0001
Refractive index, n^20^	1.4821	0.0015
Optical rotation, [*α*] (°)	+6.15	0.19
Color		
Subjective	Mustard green	
RGB color values	R: 203, G: 201, B: 111	
CMYK color values	C: 0, M: 1, Y: 45, K: 20	
Hex Color Codes	#f7f019	

**Table 2:** Chemical composition of essential oil from fruits of *Citrus* x *limonia*.

CN	RT	Compound	RIC	RIR	%			CG	CF	MM (Da)
1	9.09	*α*-Thujene	924	924	0.46	±	0.04	MH	C_10_H_16_	136.13
2	9.42	*α*-Pinene	932	932	2.39	±	0.08	MH	C_10_H_16_	136.13
3	10.18	Camphene	946	946	0.07	±	0.01	MH	C_10_H_16_	136.13
4	11.22	Sabinene	970	969	1.47	±	0.44	MH	C_10_H_16_	136.13
5	11.46	*β*-Pinene	975	974	12.04	±	0.63	MH	C_10_H_16_	136.13
6	11.95	Myrcene	989	988	1.92	±	0.06	MH	C_10_H_16_	136.13
7	12.95	*n*-Octanal	998	998	0.31	±	0.02	OC	C_8_H_16_O	128.12
8	13.29	*α*-Terpinene	1014	1014	0.57	±	0.11	MH	C_10_H_16_	136.13
9	13.78	*ο*-Cymene	1022	1022	0.47	±	0.20	MH	C_10_H_14_	134.11
10	14.00	Limonene	1024	1024	57.38	±	1.09	MH	C_10_H_16_	136.13
11	14.06	*β*-Phellandrene	1026	1025	0.53	±	0.02	MH	C_10_H_16_	136.13
12	14.23	1,8-Cineole	1028	1026	0.79	±	0.04	OM	C_10_H_18_O	154.14
13	14.72	(*E*)-*β*-Ocimene	1045	1044	0.24	±	0.01	MH	C_10_H_16_	136.13
14	15.35	*γ*-Terpinene	1055	1054	13.01	±	0.37	MH	C_10_H_16_	136.13
15	16.31	(2*Z*)-Hexenal diethyl acetal	1083	1081	tr		-	OM	C_10_H_20_O_2_	172.15
16	16.63	Terpinolene	1087	1086	0.84	±	0.12	MH	C_10_H_16_	136.13
17	17.56	Linalool	1097	1095	0.16	±	0.01	OM	C_10_H_18_O	154.14
18	17.84	*n*-Nonanal	1102	1100	0.09	±	0.01	OC	C_9_H_18_O	142.14
19	18.64	*exo*-Fenchol	1120	1118	0.03	±	0.00	OM	C_10_H_18_O	154.14
20	20.14	Citronellal	1150	1148	0.20	±	0.04	OM	C_10_H_18_O	154.14
21	21.60	Terpinen-4-ol	1176	1174	1.11	±	0.44	OM	C_10_H_18_O	154.14
22	22.40	*α*-Terpineol	1188	1186	1.21	±	0.42	OM	C_10_H_18_O	154.14
23	22.72	*n*-Decanal	1203	1201	0.21	±	0.02	OM	C_10_H_20_O	156.15
24	24.44	Neral	1237	1235	0.03	±	0.01	OM	C_10_H_16_O	152.12
25	25.84	Geranial	1266	1264	0.05	±	0.01	OM	C_10_H_16_O	152.12
26	27.43	Undecanal	1307	1305	0.03	±	0.00	OC	C_11_H_22_O	170.17
27	30.19	*α*-Copaene	1376	1374	0.10	±	0.01	SH	C_15_H_24_	204.19
28	30.73	*β*-Cubebene	1389	1387	0.05	±	0.00	SH	C_15_H_24_	204.19
29	31.91	Dodecanal	1409	1408	0.04	±	0.01	OC	C_12_H_24_O	184.18
30	32.18	(*E*)-Caryophyllene	1418	1417	0.38	±	0.06	SH	C_15_H_24_	204.19
31	32.64	*α*-*trans*-Bergamotene	1434	1432	0.66	±	0.14	SH	C_15_H_24_	204.19
32	32.84	(*Z*)-*β*-Farnesene	1440	1440	0.03	±	0.00	SH	C_15_H_24_	204.19
33	33.75	*α*-Humulene	1453	1452	0.06	±	0.01	SH	C_15_H_24_	204.19
34	34.84	Germacrene D	1482	1480	0.57	±	0.07	SH	C_15_H_24_	204.19
35	35.46	Bicyclogermacrene	1502	1500	0.07	±	0.01	SH	C_15_H_24_	204.19
36	35.66	(*E*,*E*)-*α*-Farnesene	1506	1505	0.63	±	0.18	SH	C_15_H_24_	204.19
37	35.85	*β*-Bisabolene	1507	1505	1.10	±	0.24	SH	C_15_H_24_	204.19
38	42.06	*α*-Cadinol	1654	1652	0.02	±	0.00	OS	C_15_H_26_O	222.20
39	43.17	*α*-Bisabolol	1687	1685	0.04	±	0.01	OS	C_15_H_26_O	222.20
Monoterpene hydrocarbons (MH)					91.39					
Oxygenated monoterpenes					3.78					
Sesquiterpene hydrocarbons (SH)					3.65					
Oxygenated sesquiterpene					0.06					
Other compounds					0.47					
Total identified					99.35					

CN: compound number, assigned according to their elution order; RT: retention time; RIC: calculated retention indices; RIR: retention index based on literature; %: relative abundance; CF: chemical formula; MM: monoisotopic mass; CG: compound group; tr: traces.

**Table 3:** Pairs of enantiomers separated from the essential oil of *Citrus* x *limonia* fruits using a MEGA-DEX DET Beta column.

RT	Enantiomers	RI	ED (%)	e.e. (%)
3.81	(+)-(1R,5R), (+)-(1S,5S), *α*-Pinene	931	3.92	92.15
3.90		944	96.08	
5.17	(+)-(1R,5R)-*β*-Pinene	997	85.42	70.84
5.48	(−)-(1S,5S)-*β*-Pinene	1006	14.58	
6.21	(+)-(1R,5R), (+)-(1S,5S), Sabinene	1024	58.34	16.47
6.50		1032	41.88	
7.35	(−)-(4S), (+)-(4R), Limonene	1053	1.93	96.14
7.39		1055	98.07	

RT: retention time; RI: retention index; ED: enantiomeric distribution; e.e.: enantiomeric excess.

**Table 4:** Chiral compounds present in the essential oil from fruits of *Citrus* x *limonia* determined using the MEGA-DEX DAC Beta column.

RT	Enantiomers	RI	ED (%)	e.e. (%)
30.35	(−)-(4S), (+)-(4R), α-Terpineol	1396	17.95	64.11
30.56		1399	82.05	

RT: retention time; RI: retention index; ED: enantiomeric distribution; e.e.: enantiomeric excess.

**Table 5:** Antibacterial activity of essential oil from fruits of *Citrus* x *limonia*.

Microorganism	*Citrus* x *limonia* Fruit EO	Positive Control *	Negative Control
	MIC (µg/mL)		
Gram-positive cocci			
*Enterococcus faecalis* (ATCC 19433)	>4000	0.78	+
*Enterococcus faecium* (ATCC 27270)	>4000	0.39	+
*Staphylococcus aureus* (ATCC 25923)	>4000	0.39	+
Gram-positive bacillus			
*Lysteria monocytogenes* (ATTC 19115)	>4000	1.56	+
Gram-negative bacilli			
*Escherichia coli* O157:H7 (ATCC 43888)	>4000	1.56	+
*Campylobacter jejuni* (ATCC 33560)	>4000	1.56	
*Pseudomonas aeruginosa* (ATCC 10145)	>4000	0.39	+
*Salmonella enterica* subs enterica serovar Thypimurium WDCM 00031, derived (ATCC 14028)	>4000	0.39	+

*: ampicillin for Gram-positive cocci and ciprofloxacin for Gram-positive bacillus and Gram-negative bacilli; +: normal growth.

**Table 6:** Antimicrobial activity of essential oil from fruits of *Citrus* x *limonia*.

Microorganism	*Citrus* x *limonia* Fruit EO	Positive Control *	Negative Control
	MIC (µg/mL)		
Fungi			
*Aspergillus niger* (ATCC 6275)	4000	0.098	+
Yeasts			
*Candida albicans* (ATTC 10231)	>4000	0.098	+

*: Amphotericin B; MIC: minimum inhibitory concentration; +: normal growth.

**Table 7:** Antioxidant activity of *Citrus* x *limonia* fruits essential oil.

Sample	ABTS	DPPH	TEAC
	SC_50_ ± SD (µg/mL) *		Mean ± SD (µM TE/g)
*Citrus* x *limonia* fruit EO	1261.88 ± 1.52	8145.65 ± 5.31	21.75 ± 3.75
Trolox	29.09 ± 1.05	35.54 ± 1.04	

ABTS: 2,2′-azinobis(3-ethylbenzothiazoline-6-sulfonic acid); DPPH: 2,2-diphenyl-1-picrylhydrazyl; TEAC: trolox equivalent antioxidant capacity; TE: trolox equivalent; *: µM for trolox; SD: standard deviation.

## Data Availability

Data are available from the authors upon reasonable request.
